# Preoperative oral diazepam for intraoperative blood pressure stabilisation in hypertensive patients undergoing vitrectomy under retrobulbar nerve block anaesthesia: study protocol for a randomised controlled trial

**DOI:** 10.1186/s13063-022-06686-y

**Published:** 2022-09-02

**Authors:** Tianwei Qian, Qiaoyun Gong, Chong Chen, Xia Wu, Lin Xue, Ying Fan, Weijun Wang, Zhihua Zhang, Hui Cao, Xun Xu

**Affiliations:** 1 Department of Ophthalmology, Shanghai General Hospital, Shanghai Jiao Tong University, Shanghai, China; 2 National Clinical Research Center for Eye Diseases, Shanghai, China; 3 Shanghai Key Laboratory of Ocular Fundus Diseases, Shanghai, China; 4 Shanghai Engineering Center for Visual Science and Photomedicine, Shanghai, China; 5 Shanghai Engineering Center for Precise Diagnosis and Treatment of Eye Disease, Shanghai, China

**Keywords:** Retrobulbar anaesthesia, Vitrectomy, Diazepam, Blood pressure stabilisation, Study protocol

## Abstract

**Background:**

As a type of local anaesthesia, retrobulbar nerve block is often used in vitrectomy, with patients remaining conscious during the operation. The increase in systolic blood pressure (SBP) caused by tension and fear during the operation—especially in patients with a history of hypertension—can negatively impact the safety of the procedure, resulting in suprachoroidal haemorrhage or retinal haemorrhage. Diazepam has a sedative effect and can relieve tension during surgery. This study aims to evaluate the efficacy and safety of diazepam for intraoperative BP stabilisation in hypertensive patients under retrobulbar anaesthesia during surgery.

**Methods:**

This single-centre, double-blind, randomised controlled and parallel clinical trial will include 180 hypertensive patients who will undergo vitrectomy with nerve block anaesthesia. Study participants will be randomly allocated in a 1:1 ratio to intervention (patients receiving oral diazepam before the operation) and control (patients receiving oral placebo before the operation) groups. The primary outcome is the effective rate of intraoperative BP control (systolic BP during operation maintained at <160mmHg at all timepoints). The secondary outcomes are the proportion of patients with SBP ≥180 mmHg at any timepoint from operation to 1 h post-operation, the change of mean systolic blood pressure and mean heart rate during operation from baseline, as well as the number of patients with intraoperative and post-operative adverse reactions within 12 weeks of surgery. The logistic regression model will be performed to compare the outcomes.

**Discussion:**

This study will evaluate the efficacy and safety of diazepam for intraoperative BP stabilisation in hypertensive patients under nerve block anaesthesia during surgery. The results of this trial will reveal whether diazepam has a significant effect on intraoperative BP stability in patients with a history of hypertension who require vitrectomy. If the results of this trial are significant, a large-scale multi-centre clinical trial can be designed.

**Trial registration:**

Chinese Clinical Trial Registry (ChiCTR) ChiCTR2100041772. Registered on 5 January 2021.

**Supplementary Information:**

The online version contains supplementary material available at 10.1186/s13063-022-06686-y.

## Background

Vitrectomy, a minimally invasive surgery for releasing vitreoretinal traction and restoring a transparent refractive stroma, has been widely used to treat vitreoretinal diseases including rhegmatogenous retinal detachment, traction retinal detachment, macular epiretinal membrane, and macular hole. This type of high-level modern ophthalmic microsurgery was developed in the early 1970s and is considered as revolutionary in the history of ophthalmic treatment [1]. With the ageing of the global population and increasing prevalence of vitreoretinopathy, small-gauge vitrectomy is becoming a more widely performed procedure [2]. This surgery has traditionally been performed under general anaesthesia (inhaled as a gas) to induce a reversible loss of consciousness. In contrast, local anaesthesia involves the administration of anaesthetic to a specific part of the body to induce a localised loss of sensation [3]. With the exception of patients who require general anaesthesia for personal reasons, retrobulbar nerve block is one of the most commonly used methods of local anaesthesia for vitrectomy; the anaesthetic is administered posteriorly into the retrobulbar space within the muscle cone, allowing for a longer duration of anaesthesia but also increasing ocular exposure [4, 5].

Modern retrobulbar blockade techniques have long been considered as the gold standard for anaesthesia in intraocular surgery [6]. However, the patient is awake during the operation, which can lead to stress and increased systolic blood pressure (SBP), which enhances the risk of retinal haemorrhage and cardiovascular and cerebrovascular events [7, 8]. Therefore, maintaining a stable intraoperative BP during vitrectomy is critical for patients’ safety.

Some patients who are normally nervous and prone to insomnia take diazepam tablets immediately before or on the day before the surgery to relieve intraoperative tension, which reduces fluctuations in intraoperative BP. Diazepam, also known as Valium, is a long-acting benzodiazepine. This class of drugs inhibits the central nervous system, which has the clinical effects of sedation and hypnosis; they are also used as anxiolytics and anticonvulsants [9]. Diazepam taken orally is rapidly and completely absorbed, with plasma concentrations peaking after 0.5–2 h. Diazepam is mainly metabolised in the liver and its metabolites include diazepam and demethyldiazepam. Although long-term use of benzodiazepines at therapeutic dosages can lead to dependence [10] and cognitive dysfunction [11], there are no reports of adverse reactions caused by a single dose.

The present study will investigate the efficacy and safety of diazepam on intraoperative BP stability in patients undergoing vitrectomy with retrobulbar nerve block anaesthesia. In patients with a history of hypertension, we hypothesise that oral diazepam before surgery is safe and effective in maintaining a stable BP during vitrectomy.

## Methods/design

### Overview

This is a single-centre, double-blind, parallel and randomised clinical trial (RCT) being conducted at Shanghai General Hospital affiliated with Shanghai Jiaotong University. The patients have a clear history of hypertension that is under control and require vitrectomy. Our hypothesis is that among patients with hypertension before vitrectomy, a greater proportion will have a stable BP during the operation with pre-operative diazepam vs the placebo. After comprehensive ophthalmic and physical examinations, the patients will be randomised into the diazepam or placebo group. The first participant was enrolled in August 2021. The projected completion date is August 2023.

### Objective

The study aims to evaluate the efficacy and safety of diazepam for intraoperative BP stabilisation in hypertensive patients under nerve block anaesthesia during surgery.

### Trial design

**Fig. 1:**
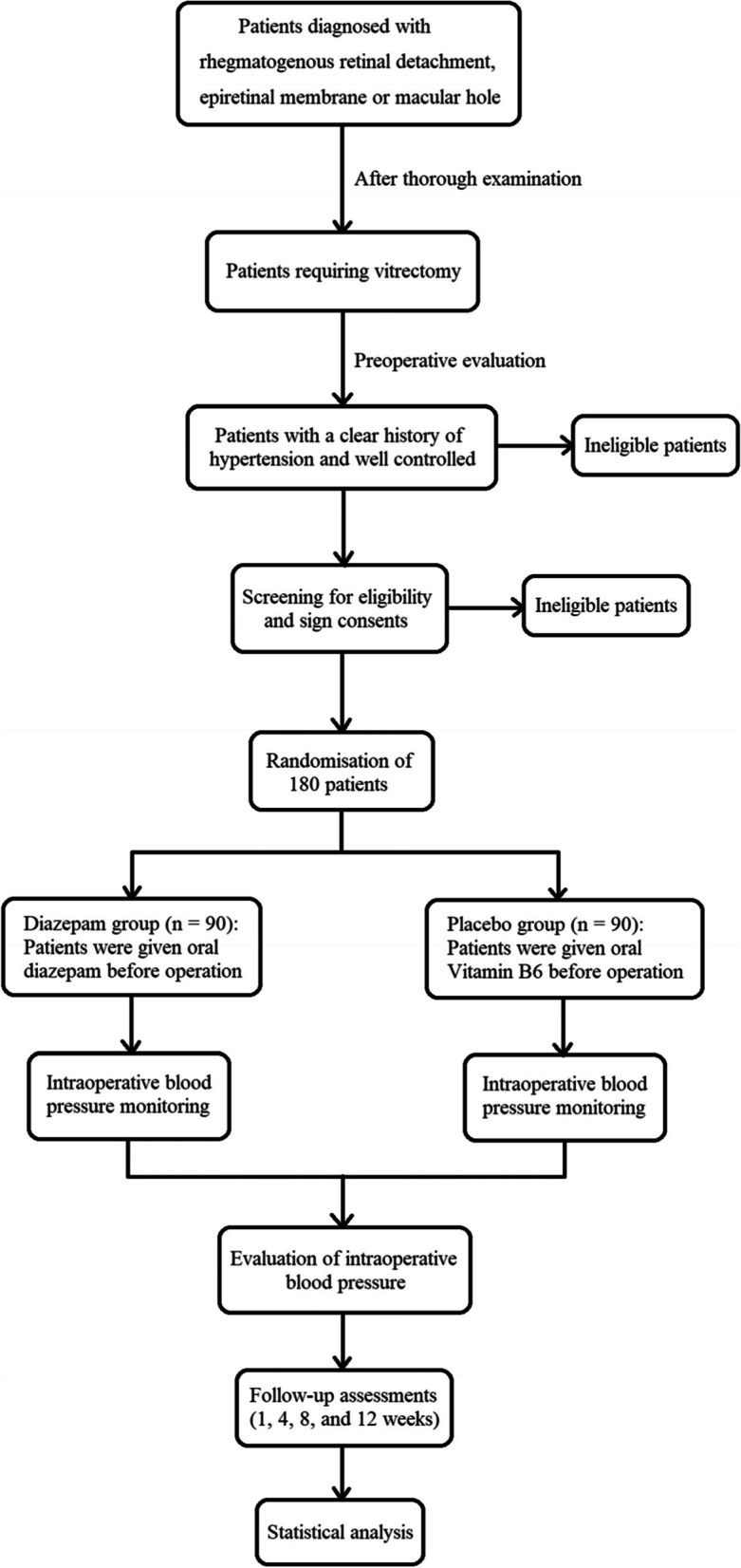
Flowchart of the trial design

This is a prospective, single-centre RCT in which patients will be allocated to two parallel groups. Vitrectomy and BP monitoring during the operation will be performed by experienced doctors. The protocol was developed in accordance with the Consolidated Standards of Reporting Trials guidelines [12, 13]. The trial has been registered with the Chinese Clinical Trial Registry (no. ChiCTR2100041772). Any protocol modifications will be communicated to participants and to the trial registry. This protocol was written in accordance with the Standard Protocol Items: Recommendations for Interventional Trials (SPIRIT) [14] guidelines (Table 1 and Additional file 1). Trial results will be disseminated through publication or presentation at scientific conferences. A flowchart of the trial design is shown in Fig. 1.

**Table 1:** Standard Protocol Items: Recommendations for Intervention Trials (SPIRIT)

Timepoint	Study period				
	Enrolment	Allocation	Perioperation	Post-surgery	Follow-up
	Recruitment (1 day before operation)	Randomisation (1h before operation)	Before and after anaesthesia, every 5 min from the start of operation	1 h after operation	Within one month after operation
**Enrolment:**					
Eligibility screen	**×**				
Informed consent	**×**				
Randomisation and allocation		**×**			
**Interventions:**					
Diazepam group (oral diazepam 2.5 mg before operation)		**×**			
Placebo group (oral placebo before operation)		**×**			
**Assessments:**					
Blood pressure	**×**	**×**	**×**	**×**	**×**
Heart rate	**×**	**×**	**×**	**×**	**×**
Adverse reactions		**×**	**×**	**×**	**×**

The RCT will enrol 180 participants who will be randomised in a 1:1 ratio to the intervention group (oral diazepam 2.5 mg before surgery) or control group (oral placebo before surgery). The patient’s history of hypertension and the first random BP measurement at admission will be recorded at the screening visit for the trial. The BP 1 h before the operation will be taken as the baseline reading. In both groups, intraoperative BP will be recorded every 5 min from the start of operation, which refers to after anaesthesia. Adverse events during treatment and at 1, 4, 8, and 12 weeks post treatment will be recorded. Participants have the right to withdraw their consent and discontinue participation at any time.

### Recruitment

Participants will be recruited by the surgeon during hospitalisation without any other form of advertising. Interested patients will be invited to discuss with a cardiologist the importance of intraoperative BP stabilisation. Participants meeting all of the inclusion criteria will be fully informed of their responsibilities and all procedures involved in the trial and will be asked to sign the written informed consent before enrolment.

### Inclusion criteria

The inclusion criteria are as follows: (1) ≥18 years old, with no history of operations; (2) a clear history of arterial hypertension, with well-controlled BP (defined as BP smaller than 140/90 mmHg after the treatment with antihypertensive drugs) in the last 6 months by oral administration of a single calcium channel blocker; (3) requiring vitrectomy under nerve block anaesthesia based on relevant examinations including slit lamp, fundus photography, ocular B ultrasound, optical coherence tomography, fundus fluorescein angiography, indocyanine green angiography combined with a clinical diagnosis of rhegmatogenous retinal detachment, epimacular membrane, or macular hole; (4) no history of diabetes or family history of diabetes, and preoperative HbA1c ≤6%; (5) disease is not self-limiting and there is no refractive medium opacity or pupil narrowing that affects the fundus examination; (6) no history of coronary heart disease, cerebrovascular disease, or drinking and smoking and coagulation function is normal; (7) except for calcium channel blockers to reduce BP, no drugs use under ordinary normal circumstances; (8) body weight between 60 and 75 kg on admission; and (9) provision of informed consent.

### Exclusion criteria

Exclusion criteria are as follows: (1) the patient voluntarily withdraws from the study or does not sign the informed consent form; (2) the patient ordinarily takes diazepam or other psychotropic drugs; (3) history of ataxia and tremor; (4) history of rash or leukopaenia; (5) allergy to benzodiazepines; (6) abnormal liver or kidney function and unstable BP; (7) insomnia, depression, central nervous system depression, or other mental diseases; (8) myasthenia gravis, attention deficit hyperactivity disorder, or chronic obstructive pulmonary disease; (9) cardiovascular events such as stroke, cerebral ischemia, or myocardial infarction within 6 months before screening; (10) existing diseases or conditions of the target eye or whole body (e.g. malignant hypertension; AIDS; malignant tumour; serious mental, cardiovascular, neurologic, respiratory, digestive, or other systemic disease; long-term use of hormones; or immunodeficiency after heart stenting or organ transplantation); (11) the patient is not using effective contraceptive measures or is planning to become pregnant within 6 months, or is pregnant or lactating; (12) the researchers decide during the course of the study that it is unsuitable for the patient to continue participating; and (13) participation in other trials.

### Randomisation and blinding

The stratified randomisation method will be adopted in allocating participants to the two groups in a 1:1 ratio using SPSS v22.0 (SPSS Inc, Chicago, IL, USA). The stratification factors are as follows: (1) the history of hypertension ≥ 5 years and (2) the history of hypertension < 5 years. Random allocation sequence made in advance will be created and sealed in sequentially numbered opaque envelopes, which can allow randomising one at a time. Another researcher will be responsible for group allocation. All researchers including the outcome assessors, statisticians, and data analysts will be blinded to group assignment, but those providing the intervention/control will be informed by necessity. Diazepam and placebo tablets will be packaged in capsules of the same colour and shape. Before the trial, researchers will be fully trained in the randomisation procedure and made aware of their individual responsibilities. The successful implementation and maintenance of the randomisation and blinding method will be validated.

### Sample size

According to previous retrospective data generated by our study group, the effective rate of intraoperative BP control is 80% in the intervention group and 61% in the control group; accordingly, the proportion of patients with intraoperative SBP ≥160 mmHg is 20% in the intervention group and 39% in the control group. According to the 1999 World Health Organization-International Society of Hypertension guidelines for the management of hypertension [15], mild hypertension is defined as SBP <160 mmHg. After oral diazepam before surgery, the proportion of SBP ≥160 mmHg is almost halved, resulting in marked reductions in the incidences of retinal haemorrhage and cardiovascular events. An a priori power analysis conducted with a two-tailed test (power=0.8) at the 5% significance level indicated that at least 88 participants were required in each group; therefore, a total of 180 participants will be recruited with 1:1 allocation.

### Interventions

Both groups will include patients with a history of hypertension and good BP control who have been followed up in the cardiology department for at least 6 months. The timeline of data collection is shown in Table 2.

**Table 2:** Blood pressure monitoring schedule

Group	On admission	1 h before operation (while taking oral diazepam/placebo)	Before anaesthesia	After anaesthesia	Every 5 min from the start of the operation				1 h after the operation
					0 min	5 min	10 min		
Diazepam	**+**	**+**	**+**	**+**	**+**	**+**	**+**	**+**	**+**
Placebo	**+**	**+**	**+**	**+**	**+**	**+**	**+**	**+**	**+**

^**+**^Blood pressure measurement

### Intervention group

Patients will be given oral diazepam 2.5 mg 60 min before the operation. Electrocardiogram (ECG) monitoring will be performed during the whole procedure and BP will be measured every 5 min during and 1 h after the operation in the same ward using the same equipment.

### Control group

Patients will be given an oral starch tablet 60 min before the operation. ECG monitoring and BP measurement will be performed as for the intervention group.

### Outcome measurements

Intraoperative BP and heart rate will be recorded every 5 min (0, 5, 10, 15 min, etc). Follow-up examinations will be performed at 1, 4, 8, and 12 weeks after surgery in both groups. Furthermore, the operation record of each enrolled patient, including the length of operation, will be recorded in detail.

### Primary outcome

The primary outcome is the effective rate of intraoperative BP control. Effective BP control during vitrectomy is defined as SBP during operation maintained at <160 mmHg at all timepoints. The strengths of SBP as an outcome measure are its accessibility, non-invasiveness, and reliability.

### Secondary outcomes

The following indicators will also be used to evaluate the effectiveness of diazepam in controlling intraoperative BP stability: (1) proportion of patients with SBP ≥180 mmHg at any timepoint from operation to 1 h post-operation; (2) The change of mean systolic blood pressure measured by patients during operation performed from baseline; (3) The change of mean heart rate measured during operation performed from baseline; and (4) the number of patients with intraoperative and post-operative adverse reactions within 12 weeks of surgery. Adverse reactions include ophthalmic adverse reactions (such as intraocular appearance, endophthalmitis, corneal edema, high intraocular pressure) and systemic adverse reactions (such as abnormal heart rate, abnormal blood pressure and some reactions related to diazepam).

### Data collection and safety monitoring board

Basic demographic information and data on the history of hypertension and ocular diseases will be collected pre-intervention. BP will be recorded before and during the operation, post intervention (1 week after vitrectomy), and at the 3-month follow-up.

The Data and Safety Monitoring Board, an independent advisory group, will assure the scientific integrity and ethical standards of the RCT and will be responsible for data evaluation during the study period. Researchers will be trained to collect good quality data, promote participant retention, and complete follow-up. Data analysts will be trained on data entry, coding, security, and storage. Statisticians will provide training on data assessment and statistical analysis.

### Safety (diazepam-related adverse events)

Long-term diazepam use is associated with adverse events such as drowsiness, dizziness, weakness, memory loss, and constipation. However, to date, there are no clinical reports of adverse reactions caused by a single dose of oral diazepam. During the trial, we will record and analyse any diazepam-related adverse events (including the type and number) in each group and the possible causes. Patients will receive appropriate intervention for any adverse events that occur during the treatment and follow-up phases. Serious adverse events will be immediately reported to the primary investigator, and affected participants will receive emergency treatment.

### Patients and public involvement

Neither the patients nor the public were involved in the design of the study, nor will they be involved in its conduct (including outcome measurements). Results excluding personal data will be made available on request.

### Statistical analysis

SPSS v22.0 will be used for data processing and analysis. Categorical variables (e.g. sex and number of patients with SBP ≥160 mmHg during the operation) are expressed as numbers and frequencies. Continuous variables (e.g. age) were expressed as the mean ± standard deviation (SD). In order to adjust for baseline BP of patients and any other variables which could have a potential impact on the outcomes, the logistic regression model will be used to analyse the primary outcome. When analysing the proportion of patients with SBP ≥180 mmHg and the number of patients with intraoperative and post-operative adverse reactions, the logistic regression model also will be used. While when comparing the change of mean systolic blood pressure and mean heart rate during operation from baseline, the *t* test will be used. All statistical tests will be two-sided with *P*<0.05 considered statistically significant.

## Discussion

This article describes the design and protocol of a study that aims to evaluate the efficacy and safety of diazepam for intraoperative BP stabilisation in hypertensive patients under nerve block anaesthesia during surgery. The trial protocol has been approved by the Shanghai General Hospital research ethics board (approval number 2020-119). All study participants will sign the written, informed consent form and will be fully informed of their responsibilities as well as the procedures in the trial. The patient consent form (Chinese version) is attached as Additional file 2, and the English translation is attached as Additional file 3. Neither the patients nor the general public are involved in the development of the research question or study design. The results of the trial without personal data will be directly communicated to participants and to the public through peer-reviewed publications and conference presentations. Standard and on-site screening are being conducted by an experienced retinal surgeon and cardiologist. Anaesthesiologists will monitor the whole vitrectomy process. The results of this RCT will reveal whether diazepam has a significant effect on intraoperative BP stability in patients with a history of hypertension who require vitrectomy. If the results of this trial are significant, a large-scale multi-centre clinical trial can be designed.

## Trial status

This study is currently in the recruitment phase. The first patient was enrolled in August 2021, and the study is expected to end in August 2023 considering the coronavirus pandemic. The protocol version is 1.0, dated 25 November 2020.

## Supplementary Information

**Additional file 1.** SPIRIT checklist.

**Additional file 2.** Patient consent form (Chinese version).

**Additional file 3.** Patient consent form (English version).

## Data Availability

Currently there is no plan for public access to the dataset of this trial. The Institutional Review Board of Shanghai General Hospital, auditing and monitoring committee can get access to the dataset during the study. The submitted manuscript is a study protocol without primary data. Further information can be obtained from the corresponding author.

## Abbreviations

BP: Blood pressure
ECG: Electrocardiogram
RCT: Randomised controlled trial
SBP: Systolic blood pressure
