# Supplementary material for: Preoperative oral diazepam for intraoperative blood pressure stabilisation in hypertensive patients undergoing vitrectomy under retrobulbar nerve block anaesthesia: study protocol for a randomised controlled trial
Source: Trials. 2022 Sep 2;23:723. doi: 10.1186/s13063-022-06686-y (PMC9437388; doi:10.1186/s13063-022-06686-y)
Supplement: Supplementary file 2 — Additional file 2. Patient consent form (Chinese version). [file 13063_2022_6686_MOESM2_ESM.pdf]

知情同意书告知页

试验名称：地西泮对神经阻滞麻醉下行玻璃体切割术的高血压患者术中血压稳定的有效性及安全性研究

研究单位：上海市第一人民医院

我们邀请您参加一项地西泮对神经阻滞麻醉下行玻璃体切割术的高血压患者术中血压稳定的有效性及安全性研究的试验。在决定参加本试验前，请您详细阅读本知情同意书，如果有任何不理解的问题，您可向负责试验的研究者或试验工作小组成员要求解释任何您不清楚的术语或资料。

一、研究背景和研究目的

1、研究背景

玻璃体切割术是一种眼后段玻璃体切割术（posterior vitrectomy），作用是切除混浊的玻璃体或切除玻璃体视网膜牵拉，恢复透明的屈光间质，促进视网膜复位，以此治疗玻璃体视网膜疾病，以恢复患者的视功能。玻璃体切割术属于微创手术，常规的麻醉方式为球后神经阻滞麻醉。在实际临床中发现，一些平时较为紧张、容易失眠的病人在术前自行口服安定时，术中的紧张情绪会得到缓解，术中血压的波动会相对小。本项目国内外无类似的临床随机对照研究，目前的回顾性研究中暂未发现有不良反应发生。

2、研究目的

探究地西泮对神经阻滞麻醉下行玻璃体切割术的高血压患者术中血压稳定的有效性及安全性

二、研究方法

1、哪些人可以参加试验？

需要在神经阻滞麻醉下行玻璃体切割术的患者，并满足以下条件：

1） ≥18 周岁无手术史、患有高血压且血压平日仅通过一种钙离子阻滞剂控制良好的患者；

2） 患者入院当天随机三次血压及术前病房血压均小于 140/90mmHg；

3） 患者在本院经相关检查（裂隙灯、眼底照相、OCT、ERG、FFA、ICGA 等）并结合临床诊断为孔源性视网膜脱离、黄斑前膜、黄斑裂孔，需要在神经阻滞麻醉

下行玻璃体切割术；

4） 无糖尿病史及糖尿病家族史、且术前糖化血红蛋白小于等于 6%；

5） 患者病程不限，无影响眼底检查的屈光介质混浊及瞳孔缩小；

6） 无冠心病史、无脑血管疾病史、无酗酒吸烟史、凝血功能正常者；

7） 签署知情同意书。

2、哪些些人不能参加试验？

1） 主动退出的受试者及不签署知情同意书者；

2） 平时已口服安定等精神类药物患者；

3） 有共济失调史、震颤史；

4） 有皮疹史，白细胞减少史；

5） 对苯二氮草类药物过敏者；

6） 肝肾功能异常者；

7） 失眠者，抑郁患者，中枢神经抑制者，及其他精神类疾病罹患者；

8） 重症肌无力患者，多动症患者，慢性阻塞性肺病患者；

9） 手术时间超过 90 分钟的患者；

10） 筛选前 6 个月内出现过脑卒中、脑缺血、心肌梗死等心血管事件；

11） 目标眼或全身存在研究者认为如果继续参与本研究可能使得受试者面临较大风险的疾病或状况（如恶性高血压，艾滋病，恶性肿瘤，严重的精神、心血管、神经、呼吸、消化等系统疾病，或长期服用激素，免疫缺陷性疾病，心脏支架术后，器官移植术后等）；

12） 未使用有效避孕措施者，6 个月内计划怀孕者，哺乳期、妊娠期妇女；

13） 研究过程中由研究者认定不适宜继续参加试验着。

3、研究概况及病例数量

本研究为一项前瞻性、双盲、随机、对照、单中心研究。入组方式：自愿。

双盲的定义：患者的入组情况对患者及研究者、手术者均未告知，仅对第三方的非盲研究者告知

安定组 90 例，安慰剂组 90 例，共计 180 例。

4、药物

安定及淀粉

试验药物为安定片，安慰剂为淀粉，两者均放入胶囊内，外形上无法区分。

5、研究程序

受试者在充分知情并签署知情同意书后即可进入筛选期；筛选期由评价研究者根据入组/排除标准评估是否入选。筛选合格的受试者方可进入研究治疗期。研究医生需要您配合实施相关检查项目，以便判断您是否符合入选和排除标准。这些检查是临床诊断和治疗这种疾病时常规的检查，您需按照正常程序进行检查，并非免费的额外检查：

在对患者进行评价后，对符合的患者进行入组，患者在术前 30 分钟口服一粒安定片或者维生素 B6 片（由非盲研究者随机发药）。手术开始后进行心电监护，每 5 分钟由护士记录血压；若术中收缩压高于 160mmHg 及 180mmHg 时将联系相关科室给予相应的处理。记录患者手术后一月内不良反应的发生情况，并给予相应治疗。

三、研究风险与获益

1、研究的风险

当您的健康状况因参加本试验而受到伤害时，研究医生会采取必要的医疗措施。若试验期间，您发生任何副作用或不适，您应该立即向研究医生报告，这是至关重要的。可能会给您其他药物来控制副作用。如果您或您的医生认为您无法耐受这些副作用，观察药物可能会完全停用。参与此研究时，您将需要提供一些个人信息，我们将采取所有必要措施确保该信息的机密性。

2、研究的获益

您的参与可能会对进一步神经阻滞麻醉下玻璃体切割术对高血压患者手术血压稳定性的探究有所帮助，从而推动医学的发展，尽可能降低眼部手术出血的风险。

四、您拥有的权利

您有权力决定是否参加本试验，如果您不能立即作出决定，您有充分的时间考虑，如有需要您可以与亲属、朋友等您信赖的人商量后，再做出决定。如果您决定不参加本试验，不会影响您与研究者及申办方的关系，您不会遭到歧视或者报复，您的待遇与权益不会受到影响。如果您决定参加本试验，如无特殊原因我们希望您能够完成试验，但您有权力在试验期间随时退出。如果您决定退出，请您能够及时告诉研究者。

试验期间，您可随时了解与本试验中与您有关的信息资料。如果您对此研究有任何疑问，或在治疗过程中出现不适，请与主诊医生联系。联系医生：钱天威 15201955168。且您有权就有关您的权利或相关风险等问题进行咨询。

五、隐私和保密

您向研究者提供的个人信息（如姓名、性别、联系方式、调查问卷等），除正常研究的需要外，还可能被以下人员或单位获知：

- 研究资助机构与本试验相关的工作人员（监查员、稽查员等）；
- 国家及地方食品药品监督管理局等行政机构。

但任何人在未得到您的许可前都不可以将您的个人信息透露给他人或其他机构，除了研究者和行政机构外，其他任何人或单位都没有权利主动和您联系关于本试验的事宜，或向您直接提供有关本试验的信息。

本次试验结果可能以学术论文的方式发表，但在任何公开发表的文件中，都不会出现您的个人信息。

六、其他

1、出现下列情况时，为了您的健康，研究者可能会未经您的同意而将您撤出本试验：

- 继续参加本试验，可能会导致您的风险大于受益；
- 您未按照研究者指导，依照研究方案参加试验；
- 试验提前终止。

2、本知情同意书一式两份，研究者和您各保存一份。

七、对试验所发生伤害的补偿

如果您的损伤是直接因为参加本试验而引起，您完全不用支付为治疗而负担的医疗费用，该费用将由研究者承担。

知情同意页

同意申明：

1、本人已仔细阅读受试者须知并了解这项试验的相关背景，研究者已就研究的特点和可能存在的不良反应向我做了详细解释，并对我的问题给予了解答。

2、我知道如果我拒绝参加此项试验，我的待遇与权益不会受到影响，在了解受试者须知的全部内容并经过充分的考虑后，我自愿参加此项试验。

3、我愿意遵守研究者的指示，依照研究方案参与试验。试验期间，我有权力随时退出，但退出前，我需要及时告诉研究者。

4、试验期间，如果出现了任何不适症状，我都会及时告诉研究者。

受试者签字：

/ /

姓名（楷书） 签名 签名日期

研究者签字：

/ /

姓名（楷书） 签名 签名日期

受试者代理人/监护人（如有）签字：

受试者不能签署本页的原因：__________

代理人/监护人与受试者的关系：__________

/ /

姓名（楷书） 签名 签名日期
