# Supplementary material for: Preoperative oral diazepam for intraoperative blood pressure stabilisation in hypertensive patients undergoing vitrectomy under retrobulbar nerve block anaesthesia: study protocol for a randomised controlled trial
Source: Trials. 2022 Sep 2;23:723. doi: 10.1186/s13063-022-06686-y (PMC9437388; doi:10.1186/s13063-022-06686-y)
Supplement: Supplementary file 3 — Additional file 3. Patient consent form (English version). [file 13063_2022_6686_MOESM3_ESM.pdf]

Informed Consent Notification Page

Trial：Preoperative oral diazepam for intraoperative blood pressure stabilization in hypertensive patients undergoing vitrectomy under retrobulbar nerve block anaesthesia: Study protocol for a randomised controlled trial

Sponsor：Shanghai General Hospital

We invite you to participate in a study: Preoperative oral diazepam for intraoperative blood pressure stabilization in hypertensive patients undergoing vitrectomy under retrobulbar nerve block anaesthesia. Before you decide to participate in this trial, please read this informed consent carefully. If you have any questions you don't understand, you can ask the researcher in charge of the trial or members of the trial working group to explain any terms or materials you don't know.

1. Research background and purpose

1.1 Research background

Vitrectomy is a kind of posterior vitrectomy. Its function is to remove the turbid vitreous or vitreoretinal traction, restore the transparent refractive stroma and promote retinal reattachment, so as to treat vitreoretinal diseases and restore the visual function of patients. Pars plana vitrectomy is a minimally invasive surgery. The conventional anesthesia is retrobulbar nerve block. In clinical practice, it is found that some patients who are usually nervous and prone to insomnia take diazepam by themselves before operation, the intraoperative tension will be relieved, and the fluctuation of intraoperative blood pressure will be relatively small. There is no similar clinical randomized controlled study at home and abroad, and no adverse reactions have been found in the current retrospective study.

1.2 Research purpose

Evaluate the efficacy and safety of diazepam for intraoperative BP stabilization in hypertensive patients under retrobulbar anaesthesia during surgery.

2. Methods

2.1 Who can take part in the trial?

Patients who need vitrectomy under nerve block anesthesia and meet the following conditions:

1） ≥18 years old, no history of operations;

2） have a clear history of arterial hypertension, and the BP of these patients can be well controlled in the last 6 months by oral administration of a single calcium channel blockers (CCB);

3） the patient is determined to require vitrectomy under nerve block anaesthesia based on relevant examinations including slit lamp, fundus photography, ocular B ultrasound, optical coherence tomography, fundus fluorescein angiography, indocyanine green angiography combined with a clinical diagnosis of rhegmatogenous retinal detachment, epimacular membrane, macular hole;

4） there is no history of diabetes or family history of diabetes, and preoperative HbA1c is ≤ 6%;

5） the disease is not self-limiting and there is no refractive medium opacity or pupil narrowing that affects the fundus examination;

6） there is no history of coronary heart disease, cerebrovascular disease, or drinking and smoking and coagulation function is normal;

7） except for calcium channel blockers, which are used to reduce blood pressure, they don't take any drugs at ordinary times;

8） the patient's weight should be between 60 kg and 75 kg on admission;

9） patient provides informed consent.

2.2 Who can't take part in the experiment?

1） the patient voluntarily quits or does not sign the informed consent form;

2） the patient ordinarily takes diazepam or other psychotropic drugs;

3） history of ataxia and tremor;

4） history of rash or leukopaenia;

5） allergy to benzodiazepines;

6） the patient has abnormal liver or kidney function and unstable BP;

7） insomnia, depression, central nervous system depression, or other mental diseases;

8） myasthenia gravis, attention deficit hyperactivity disorder, or chronic obstructive pulmonary disease;

9） cardiovascular events such as stroke, cerebral ischemia, or myocardial infarction within 6 months before screening;

10） existing diseases or conditions of the target eye or whole body (e.g., malignant hypertension; AIDS; malignant tumour; serious mental, cardiovascular, neurologic, respiratory, digestive, or other systemic disease; long-term use of hormones; or immunodeficiency after heart stenting or organ transplantation);

11） the patient is not using effective contraceptive measures or is planning to become pregnant within 6 months, or is pregnant or lactating;

12） the researchers decide during the course of the study that it is unsuitable for the patient to continue participatin；

13） participation in other trials.

2.3 Trial design and sample size

This is a prospective, single-centre RCT in which patients will be allocated to two parallel groups. Admission method: voluntary.

Definition of double-blind: patients, researchers and surgeons were not informed of their enrollment, but only the third-party non blind researchers

There were 90 cases in diazepam group and 90 cases in placebo group, a total of 180 cases.

2.4 Drugs

The test drug was diazepam tablets and the placebo was starch, both of which were put into the capsule, and the appearance could not be distinguished.

2.5 Research procedures

After fully informed and signed the informed consent, the subjects could enter the screening period; In the screening period, the evaluation researchers evaluated whether they were included or not according to the inclusion / exclusion criteria. Only the qualified subjects can enter the study treatment period. The research doctor needs your cooperation in the implementation of relevant examination items to determine whether you meet the inclusion and exclusion criteria. These examinations are routine examinations for clinical diagnosis and treatment of this disease. You need to follow the normal procedures, not free additional examinations.

After the evaluation of the patients, the patients who met the criteria were enrolled. The patients took diazepam tablets or placebo 30 minutes before operation (randomly distributed by

non blind researchers). ECG monitoring was performed after the operation, and the blood pressure was recorded by nurses every 5 minutes; If the intraoperative systolic blood pressure is higher than 160mmHg and 180mmHg, relevant departments will be contacted for corresponding treatment. Record the occurrence of adverse reactions within one month after operation, and give corresponding treatment.

3. Research risks and benefits

3.1 Research risks

When your health condition is harmed by participating in this trial, the research doctor will take necessary medical measures. If you have any side effects or discomfort during the trial, it is essential that you report to the study doctor immediately. You may be given other drugs to control side effects. If you or your doctor believes that you cannot tolerate these side effects, the observation drug may be completely discontinued. When participating in this study, you will need to provide some personal information, and we will take all necessary measures to ensure the confidentiality of the information.

3.2 Research benefits

Your participation may be helpful to further explore the stability of blood pressure in patients with hypertension undergoing vitrectomy under nerve block anesthesia, so as to promote the development of medicine and reduce the risk of bleeding in eye surgery as much as possible.

4. Your rights

You have the right to decide whether to participate in the experiment. If you can't make a decision immediately, you have enough time to consider it. If necessary, you can discuss with relatives, friends and other people you trust before making a decision. If you decide not to participate in this trial, your relationship with the researcher and the sponsor will not be affected, you will not be discriminated against or retaliated, and your treatment and rights will not be affected. If you decide to participate in this trial, we hope you can complete the trial without special reasons, but you have the right to withdraw at any time during the trial. If you decide to quit, please let the researcher know in time.

During the experiment, you can know the information related to you at any time. If you have any questions about this study, or if you feel unwell during the treatment, please contact the attending doctor. Contact doctor: Qian Tianwei 15201955168. And you have the right to consult about your rights or related risks.

5. Privacy and confidentiality

The personal information (such as name, gender, contact information, questionnaire, etc.) provided by you to the researcher may be known to the following persons or units in addition to the needs of normal research:

- Staff (inspectors, inspectors, etc.) of research funding institutions related to this experiment；
- State and local food and Drug Administration and other administrative agencies.

However, no one is allowed to disclose your personal information to others or other organizations without your permission. Except for researchers and administrative organizations, no other person or organization has the right to contact you about this experiment or provide you with information about this experiment directly.

The results of this experiment may be published in the form of academic papers, but your personal information will not appear in any publicly published documents.

6. Others

6.1 In case of the following situations, the researcher may withdraw you from the trial without your consent for your health:：

- If you continue to participate in this trial, your risk may outweigh your benefit;
- You did not follow the guidance of the researcher and did not participate in the trial according to the research plan;
- Early termination of trial.

6.2 This informed consent is in duplicate, one for the researcher and one for you.

7. Compensation for injury caused by trial

If your injury is directly caused by participating in this trial, you do not have to pay the medical expenses for treatment, which will be borne by the researcher.

Informed Consent Page

Consent statement:

1. I have read the instructions to the subjects carefully and understood the relevant background of this trial. The researchers have explained the characteristics of the study and possible adverse reactions to me in detail, and answered my questions.

2. I know that if I refuse to participate in this trial, my treatment and rights will not be affected. After understanding all the contents of the instructions to the subjects and having fully considered them, I voluntarily participate in this trial.

3. I am willing to follow the instructions of the researchers and participate in the trial according to the research protocol. During the experiment, I have the right to withdraw at any time, but before I withdraw, I need to inform the researchers in time.

4. During the trial, if there are any discomfort symptoms, I will tell the researchers in time.

Signature of Subject：

/ /

Name (regular script) Signature Date

Signature of Researcher：

/ /

Name (regular script) Signature Date

Signature of agent/Guardian (if any)：

The reason why the subject can't sign this page: ____________________

The relationship between the agent/guardian and the subject: ____________________

/ /

Name (regular script) Signature Date
